# Discovery of novel bacterial topoisomerase I inhibitors by use of *in silico* docking and *in vitro* assays

**DOI:** 10.1038/s41598-018-19944-4

**Published:** 2018-01-23

**Authors:** Shayna Sandhaus, Prem P. Chapagain, Yuk-Ching Tse-Dinh

**Affiliations:** 1 Biomolecular Sciences Institute, Florida International University, Miami, FL 33199 USA; 2 Department of Chemistry and Biochemistry, Florida International University, Miami, FL 33199 USA; 3 Department of Physics, Florida International University, Miami, FL 33199 USA

## Abstract

Topoisomerases are important targets for antibacterial and anticancer therapies. Bacterial topoisomerase I remains to be exploited for antibiotics that can be used in the clinic. Inhibitors of bacterial topoisomerase I may provide leads for novel antibacterial drugs against pathogens resistant to current antibiotics. TB is the leading infectious cause of death worldwide, and new TB drugs against an alternative target are urgently needed to overcome multi-drug resistance. *Mycobacterium tuberculosis* topoisomerase I (MtbTopI) has been validated genetically and chemically as a TB drug target. Here we conducted *in silico* screening targeting an active site pocket of MtbTopI. The top hits were assayed for inhibition of MtbTopI activity. The shared structural motif found in the active hits was utilized in a second round of *in silico* screening and *in vitro* assays, yielding selective inhibitors of MtbTopI with IC_50_s as low as 2 µM. Growth inhibition of *Mycobacterium smegmatis* by these compounds in combination with an efflux pump inhibitor was diminished by the overexpression of recombinant MtbTopI. This work demonstrates that *in silico* screening can be utilized to discover new bacterial topoisomerase I inhibitors, and identifies a novel structural motif which could be explored further for finding selective bacterial topoisomerase I inhibitors.

## Introduction

Antibiotic resistance is a dire problem that is facing the global community. The emergence of drug-resistant strains of pathogenic bacteria is rendering our current antibiotics almost powerless in certain cases^1,2^, and this has grave implications for the future of public health. We are facing a “post-antibiotic era”^3^: a time where previously treatable infections, including those that may be acquired during surgeries, can become life threatening. Pathogens such as *Mycobacterium tuberculosis*, the pathogen responsible for 1.7 million deaths in 2016 alone^4^ have evolved, leaving us with multi-drug resistant tuberculosis (MDR-TB) and in some cases even extensively-drug resistant tuberculosis (XDR-TB)^5^, strains that are not responsive to many of the currently available therapies. If we are to battle the threat that is antibiotic resistance, we need novel drugs and drug targets.

Topoisomerase I is one such novel drug target that has not yet been targeted by existing antibiotic drugs^6,7^. Topoisomerases are ubiquitous enzymes that are responsible for maintaining the optimal supercoiling level of DNA inside the cell. They are important in cellular processes such as DNA replication, transcription, and repair, and maintenance of genome integrity. Bacterial topoisomerase I (TopI), a type IA topoisomerase, is specifically responsible for relaxing DNA that is negatively supercoiled. Its active-site tyrosine residue attacks the phosphodiester backbone of unwound single-stranded DNA. The enzyme thus forms a covalent phosphotyrosine bond, and creates a break in the DNA. TopI will then relax the DNA by passing the unbound strand through the break, and then sealing the nick. The DNA is released from the topoisomerase, now in a more relaxed state^8–10^. Type IA topoisomerase activity is essential for resolving topological barriers that require passage of DNA through single-strand breaks, and as such, if the topoisomerase’s activity is compromised it can lead to cell growth arrest and even cell death^7,11,12^. *M. tuberculosis* topoisomerase I is the only type IA topoisomerase present in the cell, and is essential for viability^13,14^. Loss of TopI activity in *M. tuberculosis* leads to cell death. For these reasons, bacterial topoisomerase I is a promising new drug target, especially in mycobacteria.

When it comes to drug discovery, there are many valid approaches including virtual docking, high-throughput screening, and fragment-based screening, among others^15,16^. Many of these drug discovery approaches have been used successfully to find novel structures for inhibiting specific targets. One area that has become more popular through the application of high performance computing is the use of *in silico* docking. In the docking studies, a crystal structure or homology model of the desired target is screened against a large compound library, usually hundreds of thousands of compounds^17^. The compounds are scored on their ability to interact with specific pockets on the target enzyme. Many programs are available to carry out docking studies, and combined with molecular dynamics, this method can be a powerful tool.

In these studies, bacterial topoisomerase I was the intended drug target. In this screen, the crystal structure 5D5H^18^ for *M. tuberculosis* TopI (MtbTopI) was used. This crystal structure is a truncated form of the protein (missing the last 230 residues at the C-terminal end) that retains the ability to cut and rejoin single-stranded DNA. The Elite library from Asinex was used to screen the active site region on the enzyme expected to be the DNA binding site. The compound library was first screened against the original structure, and then the top 1,000 hits from that screen were docked against molecular dynamics-generated crystal structure poses. The top hits from the virtual screen were purchased and tested in the lab. From among the most potent inhibitors, there was a shared structural motif. This discovery of a common moiety was used to fuel a second round of virtual screens, this time with available Chembridge compounds that contained the motif of interest. The *in vitro* assays results confirmed virtual screening as a worthwhile method of discovering novel bacterial topoisomerase I inhibitors, and identified a novel structural motif as a potential pharmacophore for the inhibition of MtbTopI.

## Results

### Virtual screening of Asinex Elite Library

**Figure 1:**
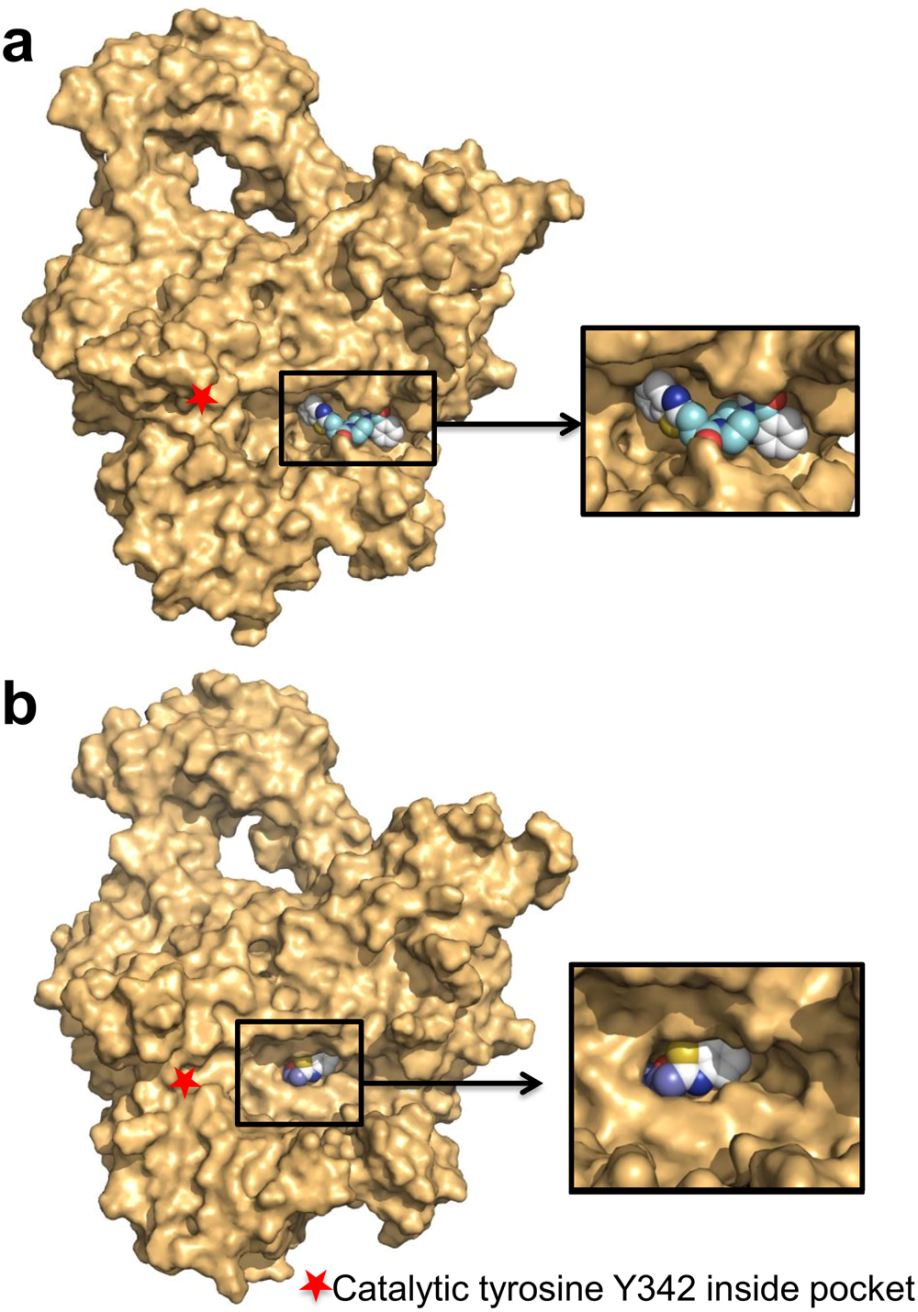
Molecular dynamics studies opened the DNA-binding pocket on MtbTopI. The Asinex compounds bind closer to the surface on the 5D5H crystal structure (**a**), while they can bind deeper inside the pocket on some of the MD-generated structures (**b**). Shown is Compound **1**.

Two screenings were carried out sequentially; the first docked the Asinex elite library of 104,000 compounds against the crystal structure 5D5H^18^, and the second docked the top 1,000 hits from the first screen against 1,000 molecular-dynamics-generated structures of 5D5H. The MD-generated structures opened the DNA-binding pocket and allowed the compounds to bind much deeper inside the pocket, as opposed to binding closer to the surface on the 5D5H crystal structure (Fig. 1). The output was used to compile a list of the top binding compounds. All of the hits were scanned using the FAF-Drugs3 program^19^ to filter out pan-assay-interference compounds (PAINS), compounds that tend to interfere with screening by non-specific interactions, thus giving “positive” results in assays of all kinds^20^. Thus ensuring none of the hits were PAINS compounds, the top 82 compounds were purchased to be tested in the lab.

### Top candidates from screening of Asinex library

The 82 purchased Asinex compounds were tested for inhibition of the relaxation activity of MtbTopI. Six compounds were found to inhibit MtbTopI with IC_50_ ≤ 500 µM (Table 1). Compound **1** (SYN 12502158) with an IC_50_ of 15.6 µM, was the most potent inhibitor against MtbTopI, with 4-fold or more selectivity for the type IA bacterial topoisomerase I versus the type IB human topoisomerase I. Compounds **2**–**4** had IC_50_ values ranging from 62.5 µM to 125 µM, and did not inhibit human topoisomerase I when tested at 250 µM.

**Table 1:** IC_50_ values of Asinex hit compounds against MtbTopI and human topoisomerase I (hTOPI).

**Compound Number**	**Asinex ID**	**MtbTopI Relaxation Inhibition (IC**_**50**_ **, µM)**	**hTOPI Relaxation Inhibition (IC**_**50**_ **, µM)**	**Selectivity Score**
**1**	SYN 12502158	15.6	93.75	6
**2**	AOP 19767246	62.5	>250	>4
**3**	ADD 15417014	62.5	>250	>4
**4**	ADM 12439418	125	>250	>2
**5**	LEG 11118762	250	n.d.	n.d.
**6**	AEM 11113320	500	n.d.	n.d.

n.d. – not determined.

Selectivity Score = hTOPI IC_50_/MtbTopI IC_50_.

### Follow-up virtual screening

**Figure 2:**
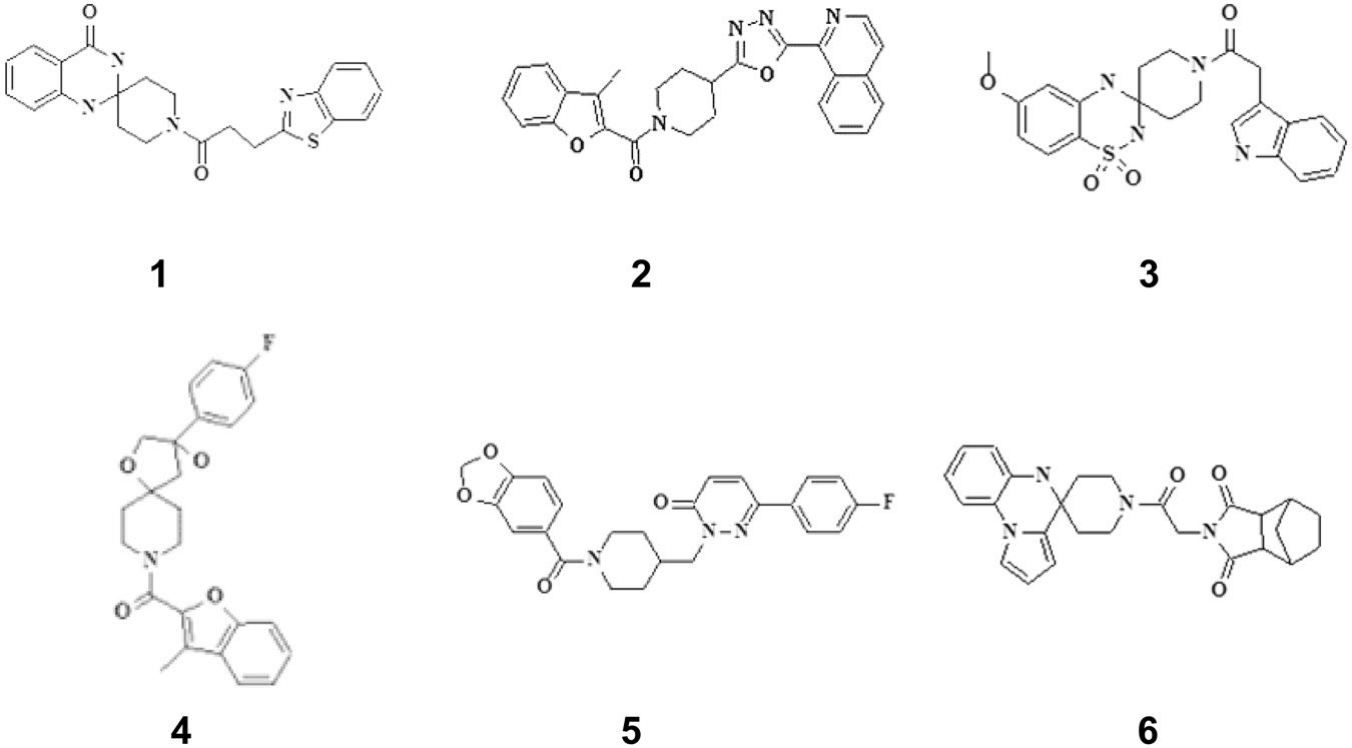
Structures of Asinex compounds identified from *in silico* screening and *in vitro* MtbTopI assay.

**Figure 3:**
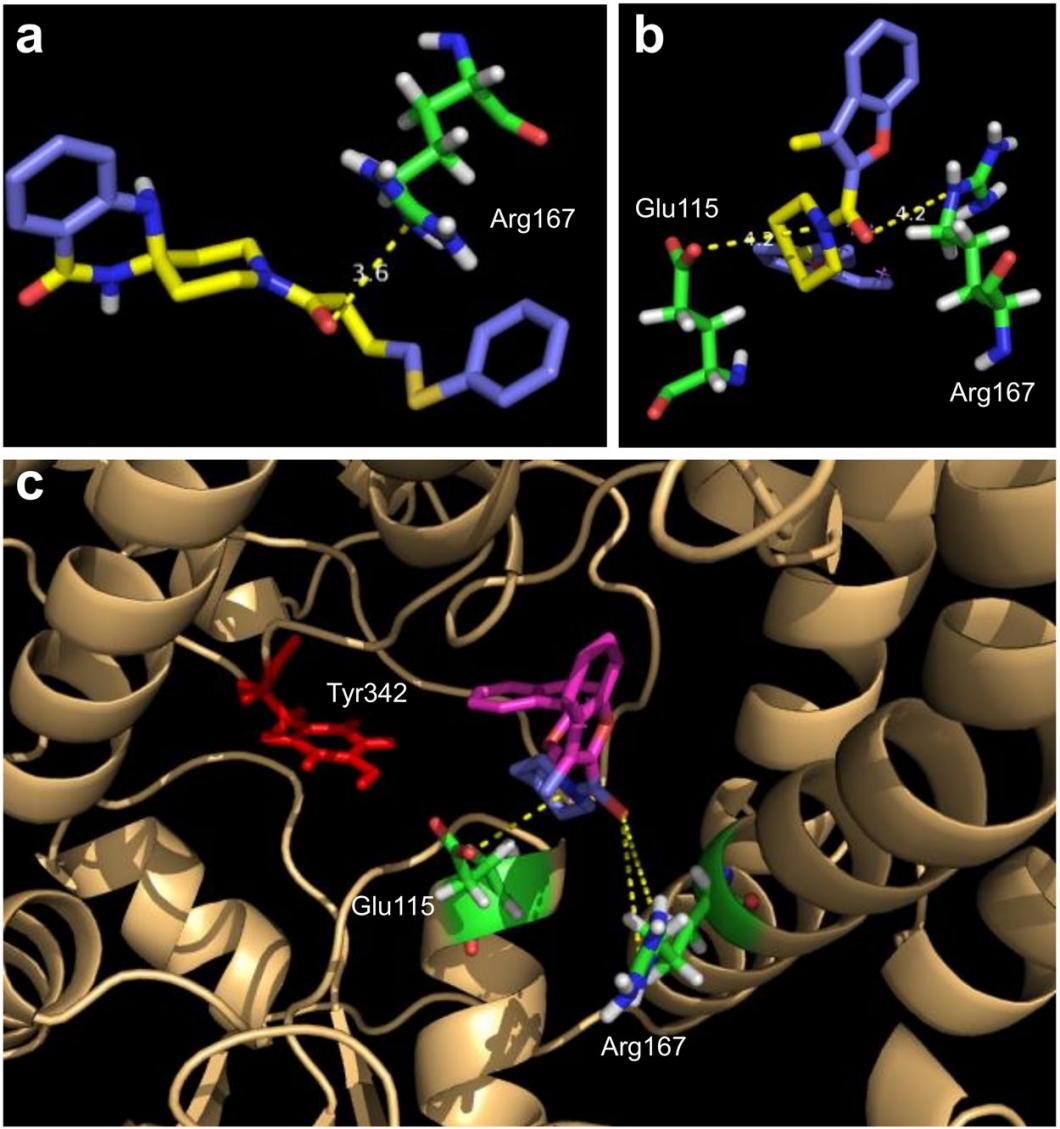
Tertiary amide moiety on Asinex hits interacts with key residues. The common piperidine amide moiety on the Asinex hits shows interactions with Arg167 and Glu115. Shown are (**a**) Compound **1** with Arg167, (**b**) Compound **2** with both Arg167 and Glu115, and (**c**) the compound’s binding position (shown is compound **2**) is located near catalytic tyrosine 342.

Although the first screen was successful at finding some MtbTopI inhibitors, there is a need to improve the potency of inhibition. Interestingly, the Asinex compounds identified contained a common structural motif—a piperidine amide located in the center of the molecule, with different groups on either side (Fig. 2). After noting this similarity, we examined the docking positions in the MD-generated structures for these top compounds to elucidate the action of this common motif. In the top docking positions, the motif appears to be interacting with key residues strictly conserved for catalysis, Arg167 and Glu115^21,22^. These residues are located in the DNA-binding pocket on the MtbTopI. The corresponding residues in *Escherichia coli* topoisomerase I can be observed to be interacting with the ribose ring of the DNA substrate in the structure of its covalent complex^22^. Specifically, the amide oxygen in the common motif interacts with Arg167, while the amide nitrogen interacts with Glu115 (Fig. 3). This structural motif may be acting as a so-called “lynchpin” to hold the compound in place at the enzyme active site. This binding behavior could explain the observed enzyme inhibition.

To further identify inhibitors of MtbTopI related to this piperidine amide motif, a search was conducted to find compounds in the Chembridge library that contained a cyclic tertiary amide motif. Over 200 compounds were found to contain such amide substructure, and they were docked with the same procedures as the Asinex compounds. The 96 compounds with the top docking scores were purchased from Chembridge for further testing.

### Top candidates from Chembridge screen inhibit bacterial topoisomerase I selectively

**Figure 4:**
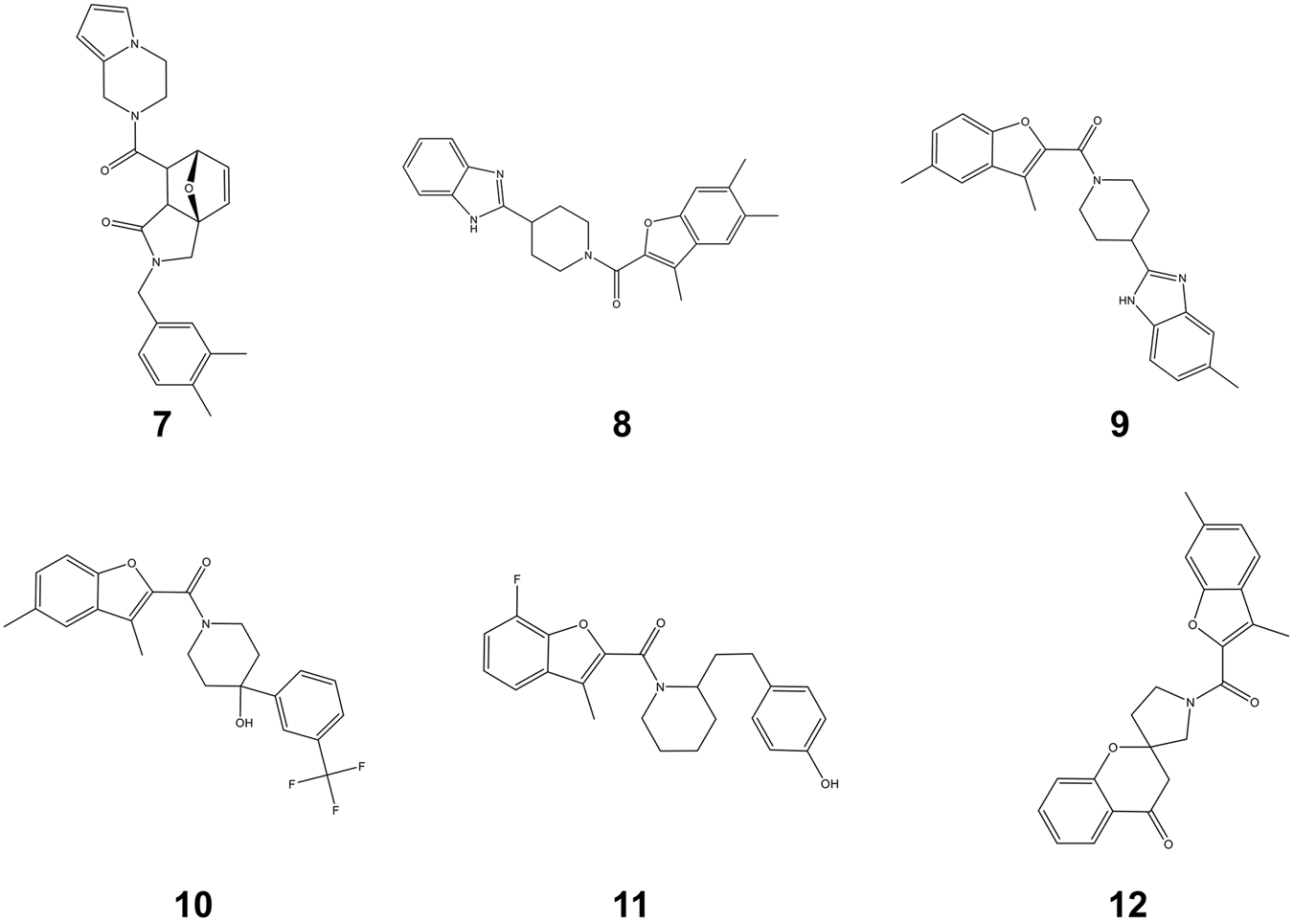
Structures of Chembridge compounds 7–12 with IC_50_ ≤ 62.5 µM.

**Figure 5:**
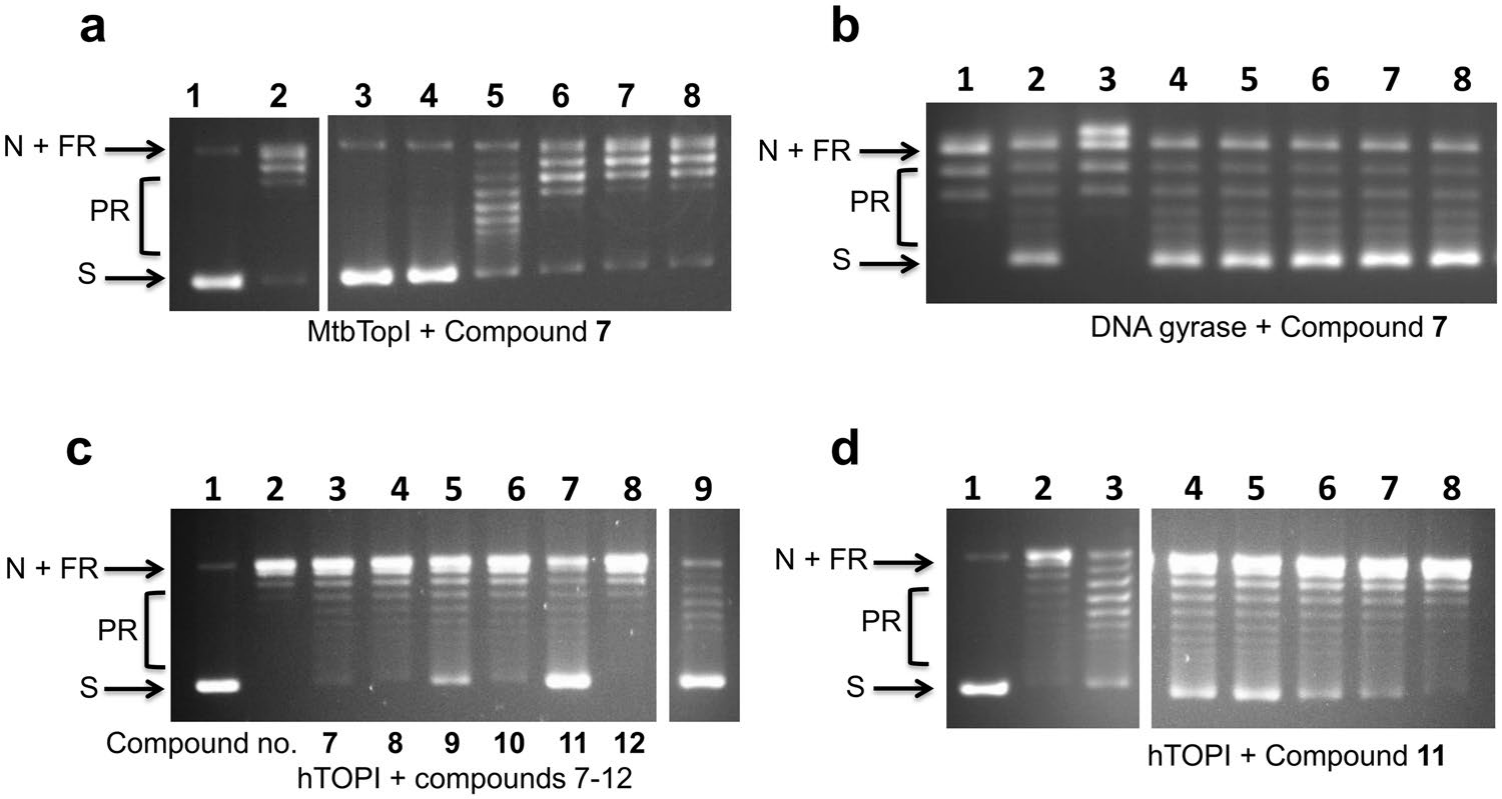
Selective inhibition of MtbTopI by Chembridge hit compounds. (**a**) Inhibition of MtbTopI relaxation activity by Compound **7**. Lane 1: negatively supercoiled plasmid DNA substrate; Lane 2: DMSO as negative control; Lanes 3–8: 8, 4, 2, 1, 0.5 and 0.25 µM Compound **7**. The lanes shown are from the same gel. (**b**) Compound **7** does not inhibit *E. coli* DNA gyrase supercoiling activity. Lane 1: relaxed covalently closed circular DNA; Lane 2: DMSO as negative control; Lane 3: 150 µM ciprofloxacin; Lanes 4–8: 500, 250, 125, 62.5, and 31.3 µM Compound **7**. (**c**) Assay of Chembridge top hits for inhibition of human topoisomerase I relaxation activity. Lane 1: negatively supercoiled plasmid DNA; Lane 2: DMSO as negative control; Lanes 3–8: Compounds **7**, **8**, **9**, **10**, **11**, and **12** respectively, at 500 µM; Lane 9: 200 µM camptothecin. Lanes 1–9 shown here are from the same gel. (**d**) Inhibition of human topoisomerase I relaxation activity by Compound **11**. Lane 1: negatively supercoiled plasmid DNA substrate; Lane 2: DMSO as negative control; Lane 3: 200 µM camptothecin; Lanes 4–8: 500, 250, 125, 62.5, and 31.3 µM Compound **11**. The lanes shown here are from the same gel. S: Supercoiled DNA, N: Nicked DNA, FR: Fully Relaxed DNA, PR: Partially relaxed DNA.

The 96 compounds purchased from Chembridge were tested *in vitro* against purified MtbTopI to ascertain their ability to inhibit enzymatic relaxation activity. Eighteen compounds were found to have IC_50_ ≤ 125 µM (Table 2). The structures of the six compounds with IC50 ≤ 62.5 µM (Compounds **7**–**12**) are shown in Fig. 4. Compound **7** (Chembridge ID 49981944) had an IC_50_ of 2 µM (Fig. 5a), significantly lower than the other compounds tested. Compound **7** was also tested against *E. coli* DNA gyrase, a bacterial type IIA topoisomerase, and no inhibition was seen at up to 500 µM, confirming the selectivity of the inhibition for type IA topoisomerase activity (Fig. 5b). Compounds **7–12** (Fig. 4) were also assayed against the human topoisomerase I and *E. coli* DNA gyrase to determine whether they are selective for type IA bacterial topoisomerase. The results showed none had IC_50_ < 250 µM for inhibition of human topoisomerase I and <500 µM for DNA gyrase (Fig. 5c,d). Gel electrophoresis of the MtbTopI and human topoisomerase I relaxation reaction products in the presence of ethidium bromide showed that the compounds did not increase the formation of nicked DNA, while camptothecin increased nicking of DNA by human topoisomerase I significantly (Supplementary Fig. S1). Assays against *E. coli* topoisomerase I (Supplementary Fig. S2) showed that Compounds **7, 8, 9** also inhibited the relaxation activity of this bacterial topoisomerase I, with Compound **7** (IC_50_ 15.6–31.3 µM) the strongest inhibitor among the three compounds tested, but with less potency than the inhibition of MtbTopI.

**Table 2:** IC_50_ values of Chembridge hit compounds against MtbTopI, human topoisomerase I (hTOPI) and *E. coli* DNA gyrase.

**Compound Number**	**Chembridge ID**	**MtbTopI Relaxation Inhibition (IC**_**50**_ **, µM)**	**hTOPI Relaxation Inhibition (IC**_**50**_ **, µM)**	***E. coli*** **DNA Gyrase Supercoiling Inhibition (IC**_**50**_ **, µM)**
7	49981944	2	>500	>500
8	9302190	62.5	>500	>500
9	37097280	62.5	>500	>500
10	88421238	62.5	>500	>500
11	73600812	62.5	250	>500
12	80760557	62.5	>500	>500
13	17951480	62.5–125	>500	n.d.
14	15044152	62.5–125	n.d.	n.d.
15	7931615	62.5–125	n.d.	n.d.
16	7875243	125	n.d.	n.d.
17	19138872	125	n.d.	n.d.
18	19046220	125	n.d.	n.d.
19	67687224	125	n.d.	n.d.
20	18538504	125	n.d.	n.d.
21	68171804	125	n.d.	n.d.
22	44982805	125	n.d.	n.d.
23	67941389	125	n.d.	n.d.
24	63920724	125	n.d.	n.d.

n.d. – not determined.

### Antibacterial assay against *M. smegmatis*

The non-pathogenic *M. smegmatis* was used in antibacterial assays to assess whether the identified MtbTopI inhibitors can inhibit the growth of mycobacteria. If inhibition of topoisomerase I catalytic activity is part of the antibacterial mode of action, the MIC should increase if recombinant MtbTopI is overexpressed^23^ (M+ strain with plasmid pTA-M+ versus the control strain Mnol with cloning vector). The overexpression of MtbTopI does not affect the general cell viability^23^. MtbTopI expression levels also do not affect the MIC of antibiotics that do not target the enzyme—ciprofloxacin MIC levels are the same in both strains. Either weak or no antibacterial activity was observed in the initial antibacterial assays, so the MIC measurement was repeated in the presence of the efflux pump inhibitor thioridazine^24,25^. The antibacterial activity for many of the compounds was enhanced by the presence of the efflux pump inhibitor. The results (Table 3) showed that the MICs for these compounds are shifted higher with the overexpression of recombinant MtbTopI, suggesting that inhibition of topoisomerase I activity contributes to the antibacterial activity. The barrier for penetrance through the mycobacterial cell wall may be the reason for lack of direct correlation between MIC and IC_50_ values.

**Table 3:** MIC values for antibacterial activity of identified MtbTopI inhibitors against *M*.

**Compound Number**	**MnoI Growth Inhibition (MIC, µM)**	**M+ Growth Inhibition (MIC, µM)**	**MnoI Growth Inhibition with TZ (MIC, µM)**	**M+ Growth Inhibition with TZ (MIC, µM)**
1	>500	>500	>500	>500
2	187.5	>250	31.3	62.5
3	250	>250	31.3	62.5
7	>500	>500	500	>500
8	>500	>500	23.4	31.3
9	125	>500	23.4	31.3
10	125	>500	23.4	46.9
11	500	>500	46.9	125
12	>500	>500	500	>500

*smegmatis* transformed with cloning vector (MnoI strain) or clone overexpressing recombinant MtbTopI (M+ strain). The efflux pump inhibitor thioridazine (TZ) at 6.25 µg/ml was included in some of the assays.

## Discussion

Multi-drug resistant TB is a serious global health challenge because of the difficulty presented for clinical treatment. There is an urgent need for new TB drugs, preferably via a novel mechanism. MtbTopI has been validated genetically and chemically to be a useful new target for TB drug discovery. MtbTopI inhibitors that can inhibit the growth of *M. tuberculosis* have been described^23,26^, but have not advanced into candidates for clinical drug development. A group of the previously described MtbTopI inhibitors was discovered by docking studies utilizing a modeled structure of MtbTopI^26,27^. With the availability of the MtbTopI crystal structure that contains the active site for DNA cleavage and rejoining, there is potential for further utilizing *in silico* screening to aid in the discovery of novel molecular scaffolds as MtbTopI inhibitors.

In this study, an active site pocket in the DNA-binding region between domains D1 and D4 of MtbTopI was targeted for *in silico* screening with the Autodock program. Molecular dynamics was used to further open the DNA-binding pocket and allow the compounds to have interactions much deeper inside the pocket. Initial docking against the Asinex Elite library identified a set of compounds with a common piperidine amide motif not found in previously characterized MtbTopI inhibitors. Examination of the docking outputs showed that the sterically rigid amide moiety may be interacting with specific Arg and Glu side chains that are strictly conserved in type IA bacterial topoisomerase I for interactions with the DNA backbone. Future structural studies of enzyme-compound co-crystal or analysis of resistant mutants selected against more potent derivatives are needed to verify the compound binding position.

When the *in silico* screening was repeated on Chembridge compounds containing a tertiary cyclic amide in their structures, a high percentage of the purchased hits were found to inhibit the relaxation activity of MtbTopI. Selectivity was maintained as confirmed by the lack of effect on type IB human topoisomerase I relaxation activity and type IIA gyrase supercoiling activity.

An MtbTopI inhibitor with an IC_50_ of 2 µM (Compound **7**) was identified among the hits from the Chembridge compounds. However, this and the other similar MtbTopI inhibitors did not show strong antibacterial activity when assayed against *M. smegmatis*. The antibacterial activity improved with the addition of an efflux pump inhibitor. The observed antibacterial activities were sensitive to the level of topoisomerase I activity in the cell. The MIC values were shifted higher with the overexpression of recombinant MtbTopI, in support of inhibition of topoisomerase I activity being at least contributing to the antibacterial mode of action.

Experimental evaluation of a set of 80 compounds with variable R group substitutions at three positions of a common polyamine scaffold in a previous study^23^ has identified small molecule inhibitors with greater potency for inhibition of MtbTopI and anti-mycobacterial activities. Future studies combining synthesis and assays of a large set of new compounds with different backbones plus substitutions can further explore the piperidine amide or cyclic tertiary amide moiety as a pharmacophore for inhibition of MtbTopI. Attempts can be made to modify this pharmacophore for improving the compound penetrance into mycobacteria, as well as enhancing the potency of inhibition. Exploration of other pockets in the MtbTopI structure by *in silico* screening may identify additional molecular scaffolds that are useful for developing specific MtbTopI inhibitors for potential clinical application as TB drugs.

## Methods

### In silico docking studies

The Asinex Elite library (http://www.asinex.com), containing just over 100,000 compounds, was screened against the crystal structure of MtbTopI truncated after the first 704 residues (pdb: 5D5H) using the AutoDock Vina 1.1.2. program^28^. The DNA-binding region in the active site was selected as the target for the screening. The compound structure files were first converted to pdbqt format, with 3-dimensional structure and added polar hydrogen atoms, using Open Babel^29^. The compounds were first screened and the resulting scores were sorted and ranked using custom scripts. The top 1,000 compounds were then selected for screening against multiple conformations of the binding site generated with molecular dynamics (MD) simulations described below. From the simulation trajectory, 1,000 conformations were selected and with 1,000 top compounds, this resulted in 1,000,000 docking runs. The scores were then sorted and the compounds were ranked according to their binding affinities.

### Molecular Dynamics Simulation

To incorporate the inherent flexibility of MtbTopI, protein conformations were generated with a 50-ns all-atom MD simulation for pdb 5D5H in explicit solvent. The system was set up using the Charmm-Gui web interface^30^. The protein was solvated with TIP3 water in cubic box and neutralized with counter ions. The solvated system (protein, water and the neutralizing ions) contained ~182,000 atoms. All-atom molecular dynamics simulations were performed with the CHARMM36 force field^31^ using NAMD 2.11^32^. The particle mesh Ewald (PME) method^33^ was used to calculate the long-range ionic interactions. The covalent bonds involving hydrogen atoms were constrained by SHAKE^34^. For each system, a 10,000-step minimization followed by 100 ps equilibration runs were performed using 1 fs time step. This was followed by the NPT (constant pressure/temperature) production runs at 300 K using 2 fs time steps for 50 ns. The pressure was controlled using the Nose-Hoover Langevin-piston method^35^, with a piston period of 50 fs and a decay of 25 fs. Similarly, the temperature was controlled using the Langevin temperature coupling with a friction coefficient of 1 ps^−1^. Visualization of the trajectories and extraction of pdb frames were done with VMD^36^.

### Mtb topoisomerase I relaxation inhibition

The relaxation inhibition assays were carried out in a buffer containing 40 mM Tris-HCl, pH 8.0, 5 mM MgCl_2_, 1 mM EDTA, and 20 mM NaCl, as described by Godbole *et al*.^26^. Briefly, 25 ng of *M. tuberculosis* topoisomerase I purified in the lab according to previous protocols^37^ was added to the reaction buffer to achieve I U/reaction mixture. The enzyme mixture was aliquoted into 10 µL before the addition of 0.5 µL of the compound of interest at various concentrations dissolved in DMSO. The mixtures were then incubated for 15 minutes at 37 °C before adding 150 ng of CsCl-gradient purified pBAD/Thio plasmid DNA in the same buffer for a final volume of 20 µL and enzyme concentration of 12.5 nM. The mixtures were further incubated at 37 °C for 30 minutes to allow for the enzyme’s relaxation activity. The reactions were stopped by the addition of 4 µL of a buffer containing 5% SDS, 0.25% bromophenol blue, and 25% glycerol. The samples were then run on a 1% agarose gel overnight at 25 V before ethidium bromide staining. IC50 was defined as the concentration of compound that resulted in 50% of the input DNA substrate remaining as supercoiled DNA in the relaxation assay. The same IC50s were observed when the experiments were replicated three times.

### Human topoisomerase I relaxation inhibition

Human topoisomerase I assays were carried out in a buffer containing 10 mM Tris-HCl, pH 8.0, 150 mM NaCl, 0.1% BSA, 0.1 mM spermidine, and 5% glycerol. The enzyme (from TopoGen) was diluted in the above buffer and aliquoted into 10 µL samples such that 0.5U was present in each. 0.5 µL of compound at various concentrations was added before the addition of 150 ng of purified pBAD/Thio purified plasmid DNA for a final volume of 20 µL. The samples were incubated for 30 minutes at 37 °C before stopping by the addition of 4 µL of buffer containing 5% SDS, 0.25% bromophenol blue, and 25% glycerol. The samples were analyzed by gel electrophoresis as previously described^23,38,39^. The experiments were replicated twice.

### DNA gyrase supercoiling inhibition assay

*E. coli* DNA gyrase was obtained from New England BioLabs. Two units of the enzyme were added to a reaction buffer provided by the manufacturer (35 mM Tris-HCl, pH 8.0, 4 mM MgCl_2_, 24 mM KCl, 2 mM DTT, 1.75 mM ATP, 5 mM spermidine, 0.1 mg/mL BSA, and 6.5% glycerol). 0.5 µL of the compounds dissolved in DMSO or the solvent alone were added to the enzyme mixture. 300 ng of relaxed covalently closed plasmid DNA was then added for a final volume of 20 µL. The reactions were incubated for 30 minutes at 37 °C before termination by the addition of 4 µL of the SDS stop buffer. The samples were then loaded into a 1% agarose gel and run at 25 V overnight^39^. The experiments were replicated twice.

### *Mycobacterium smegmatis* MIC determination

Strains used include the wild type *M. smegmatis* mc^2^155 as well its transformants containing an overexpression plasmid pTA-M+, which overexpresses MtbTopI, or the control vector pKW-noI described previously^23^. Cells were prepared by growing overnight at 37 °C in 7H9 media supplemented with 0.2% glycerol, 0.05% Tween 80, and 10% albumin, dextrose, sodium chloride (ADN). Overexpression strains were grown in the presence of 50 µg/ml hygromycin as well. The cells were grown to saturation and then diluted 1:100 in 7H9 without ADN supplementation. After another overnight growth to saturation, the cells were adjusted to OD_600_ = 0.1 and diluted 1:5 in 7H9 media. Aliquots of 50 µL of the diluted cells (corresponding to ~10^6^ CFU) were then added to a clear-bottom 96-well plate that contained 50 µL of the serially diluted compound in the same media. The plate was incubated for 48 hours with shaking at 37 °C, and the optical density was measured approximately every 4 hours. The minimum inhibitory concentration is recorded as the concentration that prevented at least 90% growth when compared to the control wells. For some of the compounds, the MIC measurements were also carried out in the presence of the efflux pump inhibitor thioridazine (from Sigma Aldrich) at 6.25 µg/ml (half the MIC for growth inhibition by thioridazine alone). The experiments were replicated three times.

### Data availability

All the relevant data are available upon request.

## Electronic supplementary material

Supplementary Information
