# Supplementary material for: Discovery of novel bacterial topoisomerase I inhibitors by use of in silico docking and in vitro assays
Source: Sci Rep. 2018 Jan 23;8:1437. doi: 10.1038/s41598-018-19944-4 (PMC5780498; doi:10.1038/s41598-018-19944-4)
Supplement: Supplementary file 1 — Supplementary Information [file 41598_2018_19944_MOESM1_ESM.pdf]

Supplementary Information

Discovery of novel bacterial topoisomerase I inhibitors by use of *in silico* docking and *in vitro* assays

Shayna Sandhaus[1,2], Prem P. Chapagain[1,3*] & Yuk-Ching Tse-Dinh[1,2*]

[1]Biomolecular Sciences Institute, Florida International University, Miami, FL 33199

[2]Department of Chemistry and Biochemistry, Florida International University, Miami, FL 33199

[3]Department of Physics, Florida International University, Miami, FL 33199

Supplementary Fig. S1. Gel electrophoresis in the presence of ethidium bromide following reactions in the presence of compounds.

Supplementary Fig. S2. Assays of inhibition of *E. coli* topoisomerase I by top hit compounds from Chembridge.

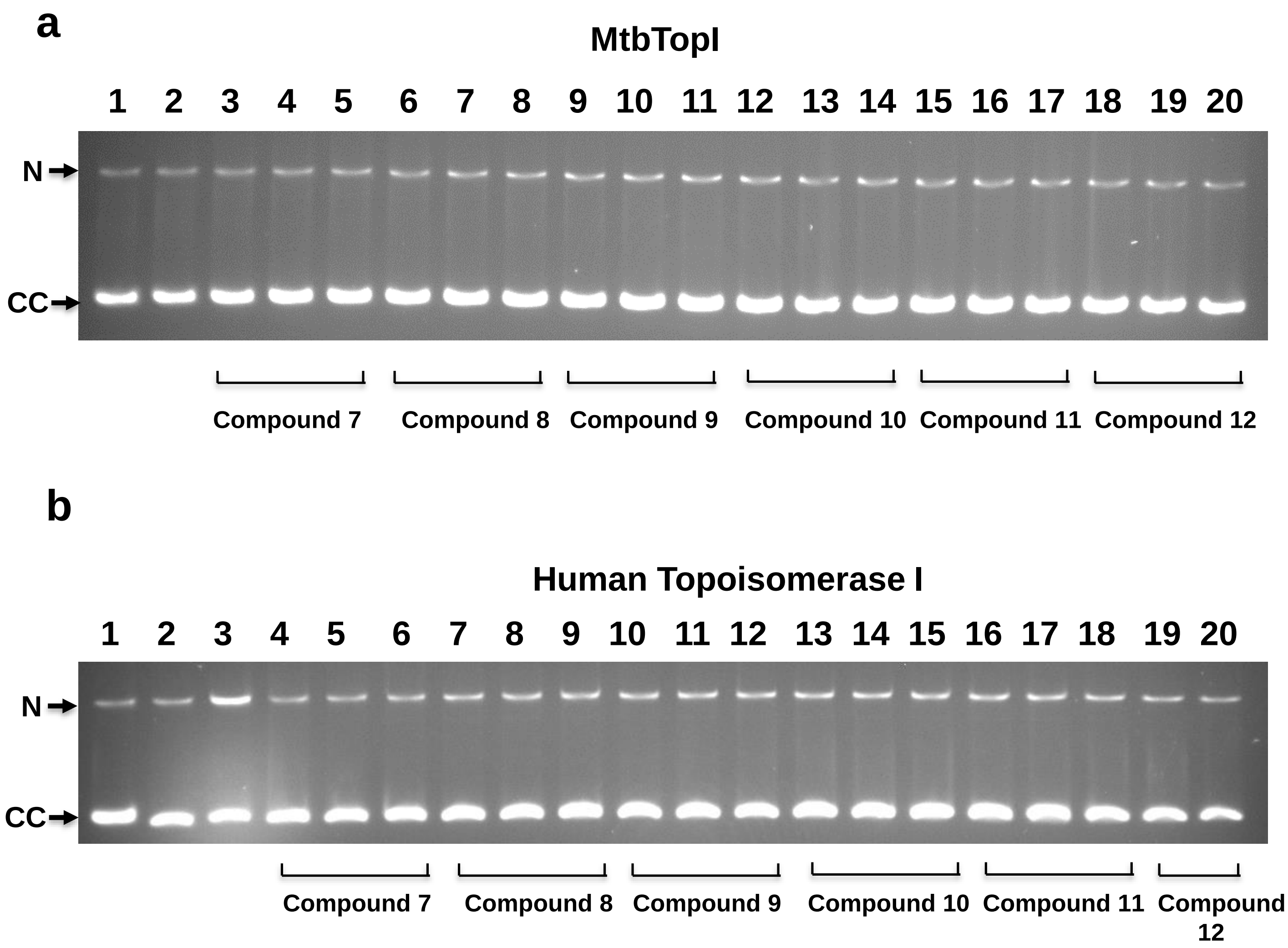

Supplementary Fig. S1. Gel electrophoresis in the presence of ethidium bromide following reactions in the presence of compounds. (a) MtbTopI reactions: Lane 1: no enzyme; Lane 2: Control with DMSO, Lanes 3-5: 8, 4, 2 μM Compound 7; Lanes 6-8: 250, 125, 62.5 μM Compound 8; Lanes 9-11: 250, 125, 62.5 μM Compound 9; Lanes 12-14: 250, 125, 62.5 μM Compound 10; Lanes 15-17: 250, 125, 62.5 μM Compound 11; Lanes 18-20: 250, 125, 62.5 μM Compound 12. (b) Human topoisomerase I reactions. Lane 1: no enzyme; Lane 2: Control with DMSO, Lane 3: 100 μM camptothecin; Lanes 4-6: 500, 250, 125 μM Compound 7; Lanes 7-9: 500, 250, 125μM Compound 8; Lanes 10-12: 500, 250, 125 μM Compound 9; Lanes 13-15: 500, 250, 125 μM Compound 10; Lanes 16-18: 500, 250, 125 μM Compound 11; Lanes 19-20: 500, 250μM Compound 12. N: Nicked DNA; CC: Covalently closed DNA.

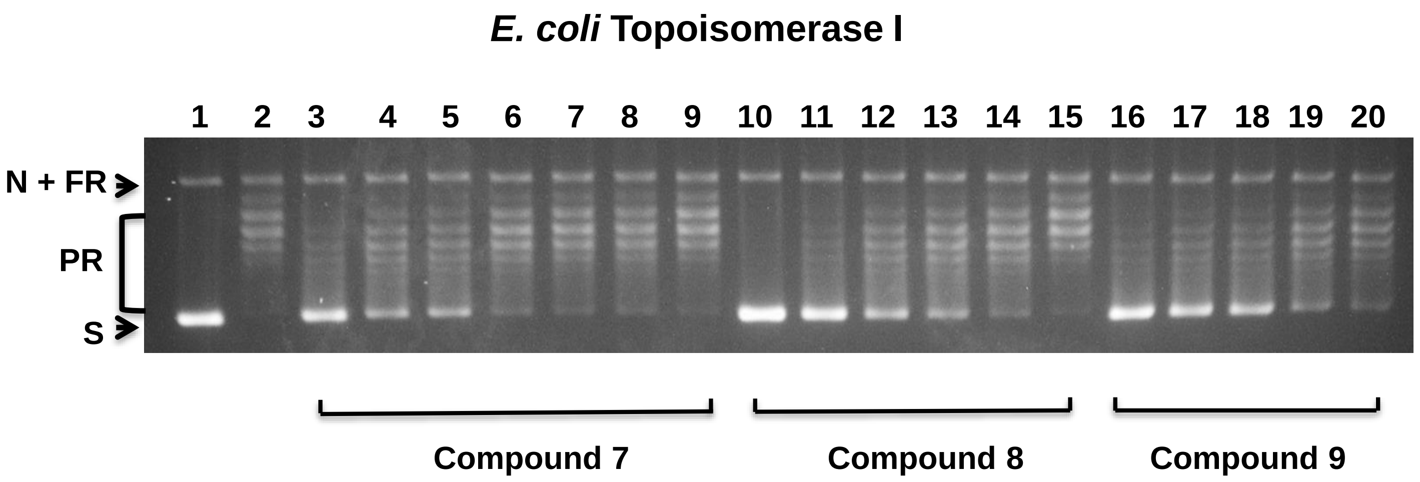

Supplementary Fig. S2. Assays of inhibition of *E. coli* topoisomerase I by top hit compounds from Chembridge. Lane 1: no enzyme; Lane 2: Control with DMSO, Lanes 3-9: 62.5, 31.3, 15.6, 8, 4, 2, 1 µM Compound 7. Lanes 10-15: 500, 250, 125, 62.5, 31.3, 15.6 µM Compound 8. Lanes 16-20: 500, 250, 125, 62.5, 31.3 µM Compound 9. N: Nicked DNA; FR: fully relaxed DNA; PR: partially relaxed DNA; S: Supercoiled DNA. Assay conditions were as described for MtbTopI. The gel was stained with ethidium bromide following electrophoresis.
